# University Education in Wales: A Royal Commission Appointed

**Published:** 1916-04-22

**Authors:** 


### UNIVERSITY EDUCATION IN WALES.

### A Royal Commission Appointed.

We are in a position to announce that the King has appointed a Royal Commission "to inquire into the organisation and work of the University of Wales and its three constituent Colleges, and into the relations of the University to those Colleges and to other institutions in Wales providing education of a post-secondary nature, and to consider in what respects the present organisation of University education in Wales can be improved, and what changes, if any, are desirable in the constitution, functions, and powers of the University and its three Colleges."

Opinion in Wales was very much divided as to the necessity of such a Commission, and the announcement of the *personnel* has therefore failed to evoke enthusiasm in the Principality.

The Welsh University Education Conference, which met several times last year, comprised as it was of representatives of the University of Wales, the Colleges of Bangor, Aberystwith, and Cardiff, and King Edward VII.'s Hospital, decided that in the midst of a great war it was undesirable to have such an inquiry; and that it would be wiser to defer such serious and important matters as the reorganisation of its University and educational methods until after the war, and when the nation had returned to its normal life.

But the Treasury was obdurate, and as the demand for increased grants for the University Colleges—reiterated over a period of years—was made dependent upon this inquiry, and the call for aid on behalf of "The Welsh National School of Medicine" at Cardiff was made dependent upon holding this Commission, the Colleges had no alternative but to agree to its challenge, and already its acceptance has ensured increased Treasury grants.

The opinion expressed that the experts appointed should be men outside the Welsh Universities, and yet acquainted with the Welsh system of education, has been met by the Government in the appointment of such men as Sir Henry Jones, LL.D., D.Litt., and the Hon. W. N. Bruce, C.B., while the appointment of Sir William Osler, who has shown great interest in the project for a Welsh School of Medicine for many years past, has given the greatest satisfaction both at Cardiff and throughout the Principality. The other members of the Commission are: The Right Hon. Viscount Haldane of Cloan, O.M., K.T., F.R.S., LL.D. (Chairman); Professor W. H. Bragg, F.R.S., M.A., D.Sc., Quain Professor of Physics in the University of London; Sir Owen M. Edwards, M.A., Chief Inspector of the Welsh Department of the Board of Education; W. H. Hadow, Esq., M.A., D.Mus., Principal of Armstrong College, Newcastle; A. D. Hall, Esq., M.A., F.R.S., a Commissioner under the Development Act; Miss Emily Penrose, M.A., Principal of Somerville College, Oxford; Secretary, Mr. A. H. Kidd, of the Board of Education.

This subject seems to have been imperfectly understood in high quarters, and perhaps this has been the main cause of the serious delay, and the chief obstacle in the completion of the Welsh Medical School. But with the appointment of Sir William Osler we are sure that all the difficulties will vanish; for his extensive experience of advanced medical education will ensure in the immediate future, and with no further loss of time, the establishment in Wales of what is certain to be the best and most modern system of higher medical education.

